# Global prevalence of prolonged gastrointestinal symptoms in COVID-19 survivors and potential pathogenesis: A systematic review and meta-analysis

**DOI:** 10.12688/f1000research.52216.1

**Published:** 2021-04-19

**Authors:** Fauzi Yusuf, Marhami Fahriani, Sukamto S. Mamada, Andri Frediansyah, Azzaki Abubakar, Desi Maghfirah, Jonny Karunia Fajar, Helnida Anggun Maliga, Muhammad Ilmawan, Talha Bin Emran, Youdiil Ophinni, Meutia Rizki Innayah, Sri Masyeni, Abdulla Salem Bin Ghouth, Hanifah Yusuf, Kuldeep Dhama, Firzan Nainu, Harapan Harapan

**Affiliations:** 1 Division of Gastroenterohepatology, Department of Internal Medicine, School of Medicine, Universitas Syiah Kuala, Banda Aceh, Aceh, 23111, Indonesia; 2 Division of Gastroenterohepatology, Department of Internal Medicine, Dr. Zainoel Abidin Hospital, Banda Aceh, Aceh, 23126, Indonesia; 3 Medical Research Unit, School of Medicine, Universitas Syiah Kuala, Banda Aceh, Aceh, 23111, Indonesia; 4 Faculty of Pharmacy, Hasanuddin University, Makassar, South Sulawesi, 90245, Indonesia; 5 Research Division for Natural Product Technology (BPTBA), Indonesian Institute of Sciences (LIPI), Wonosari, 55861, Indonesia; 6 Brawijaya Internal Medicine Research Center, Department of Internal Medicine, Faculty of Medicine, Universitas Brawijaya, Malang, East Java, 65145, Indonesia; 7 Faculty of Medicine, Universitas Brawijaya, Malang, East Java, 65117, Indonesia; 8 Department of Pharmacy, BGC Trust University Bangladesh, Chittagong, 4381, Bangladesh; 9 Ragon Institute of MGH, MIT and Harvard, Cambridge, MA, 02139, USA; 10 YARSI Hospital, Jakarta, Indonesia; 11 Department of Internal Medicine, Faculty of Medicine and Health Sciences, Universitas Warmadewa, Bali, Indonesia; 12 Department of Internal Medicine, Sanjiwani Hospital, Bali, Indonesia; 13 Department of Community Medicine, Hadhramout University College of Medicine, Mukalla, Yemen; 14 Ministry of Public Health and Population, Sana'a, Yemen; 15 Department of Pharmacology, School of Medicine, Universitas Syiah Kuala, Banda Aceh, Aceh, 23111, Indonesia; 16 Division of Pathology, ICAR-Indian Veterinary Research Institute, Izatnagar, Bareilly, Uttar Pradesh, 243122, India; 17 Department of Microbiology, School of Medicine, Universitas Syiah Kuala, Banda Aceh, Aceh, 23111, Indonesia; 18 Tropical Disease Centre, School of Medicine, Universitas Syiah Kuala, Banda Aceh, Aceh, 23111, Indonesia

**Keywords:** COVID-19, prolonged symptom, long-term effect, gastrointestinal, systematic review

## Abstract

**Background:** This study aimed to determine the cumulative prevalence of prolonged gastrointestinal (GI) symptoms, including nausea, vomiting, diarrhea, lack of appetite, abdominal pain, and dysgeusia, in survivors of both mild and severe COVID-19 worldwide and to discuss the potential pathogenesis.

**Methods:** Three databases (PubMed, Scopus, and Web of Science) were searched for relevant articles up to January 30, 2021. Data on study characteristics, clinical characteristics during follow-up, the number of patients with prolonged GI symptoms, and total number of COVID-19 survivors were retrieved according to PRISMA guidelines. The quality of eligible studies was assessed using the Newcastle-Ottawa scale. The pooled prevalence of specific prolonged GI symptoms was calculated and the association between COVID-19 severity and the occurrence of prolonged GI symptoms was assessed if appropriate.

**Results: **The global prevalence of prolonged nausea was 3.23% (95% CI: 0.54%–16.53%) among 527 COVID-19 survivors. Vomiting persisted in 93 of 2,238 COVID-19 survivors (3.19%, 95% CI: 1.62%–6.17%) and prolonged diarrhea was found in 34 of 1,073 survivors (4.12%, 95% CI: 1.07%–14.64%). A total of 156 patients among 2,238 COVID-19 survivors (4.41%, 95% CI: 1.91%–9.94%) complained of persistent decreased or loss of appetite. The cumulative prevalence of prolonged abdominal pain was 1.68% (95% CI: 0.84%–3.32%), whereas persistent dysgeusia was identified in 130 cases among 1,887 COVID-19 survivors (7.04%, 95% CI: 5.96%–8.30%). Data was insufficient to assess the relationship between COVID-19 severity and the occurrence of all prolonged GI symptoms.

**Conclusion: **Persistent GI symptoms among COVID-19 survivors after discharge or recovery raises a concern regarding the long-term impact of the COVID-19 infection on the quality of life of the survivors. Despite several potential explanations proposed, studies that aim to follow patients after recovery from COVID-19 and determine the pathogenesis of the prolonged symptoms of COVID-19 survivors are warranted.

PROSPERO registration: CRD42021239187.

## Introduction

The coronavirus disease 2019 (COVID-19), caused by severe acute respiratory syndrome coronavirus 2 (SARS-CoV-2), was initially confirmed in late December 2019 in Wuhan, China, and spread quickly globally and has become a global pandemic. As of February 6, 2021, over one hundred million confirmed cases worldwide and more than 2.5 million deaths have been reported. ^1^ COVID-19 has affected both the healthcare system ^2^^–^^5^ and socioeconomics ^6^^,^^7^ across the globe. Numerous treatment-related drug proposals ^8^^–^^10^ and vaccine development programs ^11^^,^^12^ for COVID-19 continue to be investigated, despite the many unknowns. There are no official drugs for COVID-19 that are recommended by the World Health Organization (WHO) with some treatments recommended solely on the basis of clinical trials.

The SARS-CoV-2 infection mainly affects the respiratory system, however, various other organs can also be affected ^13^^–^^17^ and with many unknown outcomes. Several studies have been conducted to assess the effects of SARS-CoV-2 on several affected health outcomes including those of the hepatic, ^18^ cardiovascular, ^19^^,^^20^ and central nervous systems, ^21^^,^^22^ and the occurrence of anosmia and dysgeusia, ^23^ as well as hemorrhagic and ischemic stroke. ^24^ Recently, gastrointestinal (GI) problems have emerged in patients with COVID-19, in particular diarrhea. ^25^ SARS-CoV-2 has been found in infected feces ^26^^,^^27^ and contaminated water supply. ^28^^,^^29^ A study reported the detection of the SARS-CoV-2 in the stool of 54% of infected patients. ^30^ The first connection of COVID-19 with GI problems was established in patients with COVID-19 in Wuhan, Hubei Province, China. ^31^ Patients with GI problems were required to stay at the hospital longer than those without GI problems. ^31^ New cases of GI symptoms have also been found in western countries. Cohort reports from the USA showed that approximately 60% of 318 patients had GI symptoms. ^32^ In the United Kingdom, a report showed that eight children with COVID-19 had atypical appendicitis symptoms. ^33^

Recent evidence suggests that the GI symptoms in patients with COVID-19 could be persistent. ^34^^,^^35^ A study in the USA found that 87.4% of patients who had recovered from COVID-19 reported persistence of at least one symptom including GI symptoms. ^34^ However, the magnitude of this persistent or prolonged occurrence of GI symptoms in those who have recovered from COVID-19 (survivors) is missing in the literature. The pathogenesis mechanisms of prolonged GI symptoms in SARS-CoV-2 infection are also scarce. In general, GI problems are accompanied by intestinal damage or inflammation. ^36^ The loss of barrier integrity in the intestine results in the invasion of microbes that could induce adaptive and immune cells, including dendritic cells. ^37^^,^^38^ However, the pathogenesis of GI problems in COVID-19 needs to be elucidated to inform better prevention and treatment approaches. The objective of this systematic review and meta-analysis was (a) to determine the global prevalence of prolonged GI symptoms including nausea, vomiting, diarrhea, lack of appetite, abdominal pain, and dysgeusia in those who had recovered from mild and severe COVID-19 and (b) to determine the association of COVID-19 severity with prolonged GI symptoms. In addition, the potential pathogenesis of these GI symptoms is also discussed.

## Methods

### Registration and protocol

The protocol of this study was registered in PROSPERO (CRD42021239187) and the protocol required no ethical clearance. To ensure the robustness of the generated data, we followed the Preferred Reporting Items for Systematic Reviews and Meta-analyses (PRISMA) guidelines to search electronic databases and report our findings. ^39^ The completed PRISMA checklist is presented in Figshare. ^40^

### Eligibility criteria of studies

Studies reporting at least one prolonged or persistent GI symptom such as nausea, vomiting, diarrhea, lack or loss of appetite, abdominal pain, and dysgeusia in patients with COVID-19 after being discharged from hospital were considered eligible. Editorials, commentaries, reviews, case reports, and case series were excluded. Diagnosis of COVID-19 must have been confirmed using RT-PCR of SARS-CoV-2 RNA from nasal or oropharyngeal swab samples. Studies that diagnosed patients with COVID-19 based on symptoms only (without nucleic acid testing) were excluded. COVID-19 survivors were defined as all patients with COVID-19 who met either the WHO or China National Health Commission discharge criteria. ^41^^,^^42^ Prolonged GI symptoms were defined as persistence of symptoms for at least two weeks after discharge in COVID-19 survivors.

### Information sources and search strategy

The potential articles in three databases (PubMed, Scopus, and Web of Science) were searched as of January 30, 2021. The searches were limited to 2019-2021 and only articles written in English were considered eligible. The search strategies were as follows. PubMed ([Title](“SARS-CoV-2” OR “COVID-19” OR “Wuhan coronavirus” OR “Wuhan virus” OR “novel coronavirus” OR “nCoV” OR “severe acute respiratory syndrome coronavirus 2” OR “coronavirus disease 2019” OR “2019-nCoV” OR “2019 novel coronavirus” OR “SARS 2”) AND ([Title](“prolong*” OR “follow-up” OR “persistent” OR “sequelae” OR “consequen*” OR “prospective” OR “cohort” OR “long-term” OR “follow*” OR “longitudinal”). Web of Science ([Title](“SARS-CoV-2” OR “COVID-19” OR “Wuhan coronavirus” OR “Wuhan virus” OR “novel coronavirus” OR “nCoV” OR “severe acute respiratory syndrome coronavirus 2” OR “coronavirus disease 2019” OR “2019-nCoV” OR “2019 novel coronavirus” OR “SARS 2”) AND ([Title](“prolong*” OR “follow-up” OR “persistent” OR “sequelae” OR “consequen*” OR “prospective” OR “cohort” OR “long-term” OR “follow*” OR “longitudinal”). Scopus ([Title](“SARS-CoV-2” OR “COVID-19” OR “Wuhan coronavirus” OR “Wuhan virus” OR “novel coronavirus” OR “nCoV” OR “severe acute respiratory syndrome coronavirus 2” OR “coronavirus disease 2019” OR “2019-nCoV” OR “2019 novel coronavirus” OR “SARS 2”) AND ([Title](“prolong*” OR “follow-up” OR “persistent” OR “sequelae” OR “consequen*” OR “prospective” OR “cohort” OR “long-term” OR “follow*” OR “longitudinal”).

### Study selection and data extraction

Essential information of all articles was imported to a reference manager (EndNote X9, Thompson Reuters, Philadelphia, PA, USA) and duplicated records among the three databases were removed. The titles and abstracts of all records were screened to identify eligible articles. The full texts of potentially eligible studies were downloaded and reviewed by two authors (MF and HH). The eligibility of each study was decided based on the eligibility criteria and the availability of the data. Data extraction was conducted as explained in previous studies. ^23^^,^^24^^,^^43^ Briefly, important data from the eligible articles were extracted and whenever required supplementary materials were extracted. The list of references was retrieved to search for additional relevant studies. The collected study characteristics of the eligible articles included author(s), year of study, study site and country, study design, extent of follow-up conducted after discharge, number of patients with COVID-19, number of patients with COVID-19 with prolonged specific GI symptoms, and severity of the COVID-19 infection during admission to the hospital.

### Outcomes

Two main outcomes were evaluated in this study: (a) global prevalence of prolonged GI symptoms including nausea, vomiting, diarrhea, lack of appetite, abdominal pain, and dysgeusia and (b) association of COVID-19 severity with the presence of prolonged GI symptoms (nausea, vomiting, diarrhea, lack of appetite, abdominal pain, and dysgeusia). In addition, the possible pathogenesis mechanisms of GI symptoms in COVID-19 including those with prolonged GI symptoms are discussed.

### Data synthesis

The prevalence of each prolonged GI symptom (nausea, vomiting, diarrhea, lack of appetite, abdominal pain, and dysgeusia) was calculated as the number of patients with a prolonged symptom divided by the total number of patients with COVID-19 with or without the specific GI symptom during the follow-up and expressed as frequency (%) with a 95% confidence interval (CI). The associations of COVID-19 severity and the risk of prolonged GI symptoms were also calculated. Forest plots were used to visualize the data.

### Risk of bias assessment

The Newcastle-Ottawa scale (NOS) was used for critical assessment of the quality of each included study. ^44^ The NOS evaluates nine characteristics of a study including four, one, and three items for sample selection, group comparison, and the outcome, respectively. The scores range between 0 to 9 and a study is classified into one of three groups based on the score: low (≤ 4), moderate (between 5–6), and high-quality (≥ 7) study.

### Statistical analysis

The Q test was used to evaluate the heterogeneity of the pooled data and the data was analyzed using a random-effect or fixed-effect model as appropriate. Egger’s test was used to assess publication bias (a *p* < 0.05 is considered indicative of potential publication bias). The associations between severity of COVID-19 and the risk of GI symptoms were calculated using the Z test. Review Manager version 5.3 was used to analyze the data. ^45^

## Results

### Study eligibility results

**Figure 1.**
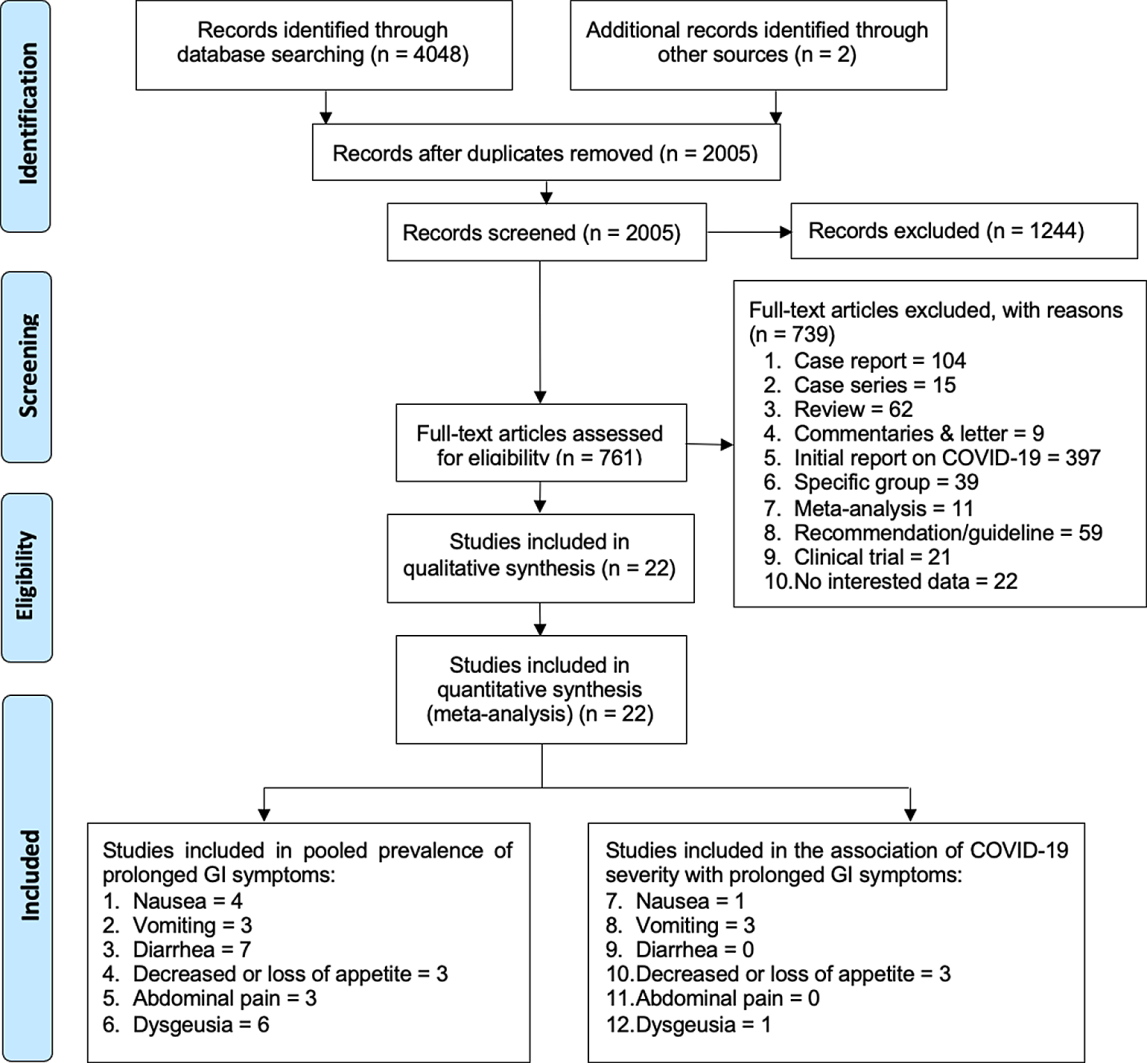
Flowchart of the result of literature search according to the Preferred Reporting Items of Systematic Reviews and Meta-Analyses (PRISMA).

The database searches yielded 4,050 eligible original articles, with 2,005 publications remaining after the duplicates were removed. Initial screening of the titles and abstract excluded 1,244 articles, leaving 761 studies ( Figure 1). After reviewing the full-texts of these studies, an additional 739 articles were excluded for several reasons including that they were reviews, case series, case reports, initial reports on COVID-19, letters or commentaries, studies on specific groups, recommendations, clinical trials, and studies with insufficient data. The final screening resulted in 22 articles which were included in this meta-analysis.

Among the 22 studies selected, the meta-analysis to calculate the prevalence of prolonged GI symptoms included four studies for nausea, ^35^^,^^46^^–^^48^ three studies for vomiting, ^35^^,^^49^^,^^50^ seven studies for diarrhea, ^35^^,^^46^^–^^48^^,^^50^^–^^52^ three studies for loss of or decrease in appetite, ^35^^,^^49^^,^^50^ three studies for abdominal pain, ^46^^,^^47^^,^^50^ and six studies for dysgeusia. ^46^^,^^47^^,^^49^^,^^53^^–^^55^ The studies included for the prevalence of prolonged GI symptoms are summarized in Table 1.

**Table 1.** The prevalence of prolonged gastrointestinal symptoms among COVID-19 survivors.

Symptom	Year	Study design	City	Country	Days from discharge to follow-up	Prevalence of GI symptom over total followed survivors			Prevalence of GI symptom based on severity of COVID-19						NOS	Ref
						No patient	Total patient	Percentage	Mild- moderate	Total	Percentage	Severe	Total	Percentage		
Nausea	2020	Prospective	Wuhan	China	14	2	131	1.53	1	62	1.61	1	69	1.45	7	35
	2020	Cohort	Georgia	USA	38 (21–49)	5	26	19.23	NA	NA		NA	NA		8	46
	2020	Prospective	Aachen	Germany	56 (48–71)	2	33	6.06	NA	NA		NA	NA		8	47
	2020	Prospective	Wuhan	China	14	1	337	0.30	NA	NA		NA	NA		7	48
	Total					10	527	1.90	1	62	1.61	1	69	1.45		
Vomiting	2021	Cohort	Hubei	China	153 (146–160)	80	1655	4.83	75	1538	4.88	5	117	4.27	8	49
	2020	Prospective	Isfahan	Iran	28	12	452	2.65	11	400	2.75	1	52	1.92	9	50
	2020	Prospective	Wuhan	China	14	1	131	0.76	1	62	1.61				7	35
	Total					93	2238	4.16	87	2000	4.35	6	169	3.55		
Diarrhea	2020	Prospective	Isfahan	Iran	28	1	452	0.22	NA	NA		NA	NA		9	50
	2020	Prospective	Wuhan	China	14	1	131	0.76	NA	NA		NA	NA		7	35
	2020	Prospective	Aachen	Germany	56 (48–71)	3	33	9.09	NA	NA		NA	NA		8	47
	2020	Prospective	Wuhan	China	14	4	337	1.19	NA	NA		NA	NA		7	48
	2020	Cohort	Georgia	USA	38 (21–49)	3	26	11.54	NA	NA		NA	NA		8	46
	2020	Prospective	Wuhan	China	??	2	18	11.11	NA	NA		NA	NA		9	51
	2020	Prospective	Wuhan	China	90	20	76	26.32	NA	NA		NA	NA		8	52
	Total					34	1073	3.17								
Loss pf appetite	2021	Cohort	Hubei	China	153 (146–160)	138	1655	8.34	127	1538	8.26	11	117	9.40	8	49
	2020	Prospective	Wuhan	China	14	3	131	2.29	1	62	1.61	2	69	2.90	7	35
	2020	Prospective	Isfahan	Iran	28	15	452	3.32	13	400	3.25	2	52	3.85	9	50
	Total					156	2238	6.97	141	2000	7.05	15	238	6.30		
Abdominal pain	2020	Prospective	Isfahan	Iran	28	6	452	1.33	NA	NA	NA	NA	NA		9	50
	2020	Cohort	Georgia	USA	38 (21–49)	1	26	3.85	NA	NA	NA	NA	NA		8	46
	2020	Prospective	Aachen	Germany	56 (48–71)	1	33	3.03	NA	NA	NA	NA	NA		8	47
	Total					8	511	1.57								
Dysgeusia	2021	Cohort	Hubei	China	153 (146–160)	120	1655	7.25	112	1538	7.28	8	117	6.84	8	49
	2020	Prospective	Fuyang	China	90	1	60	1.67	NA	NA		NA	NA		7	53
	2020	Cohort	Georgia	USA	38 (21–49)	1	26	3.85	NA	NA		NA	NA		8	46
	2020	Prospective	Aachen	Germany	56 (48–71)	3	33	9.09	NA	NA		NA	NA		8	47
	2020	Prospective	Henan	China	90	2	55	3.64	NA	NA		NA	NA		7	54
	2020	Prospective	Tokyo	Japan	108 (±23)	3	58	5.17	NA	NA		NA	NA		7	55
	Total					130	1887	6.89	112	1538	7.28	8	117	6.84		

The information about COVID-19 severity on admission and the occurrence of GI symptoms were provided in one article each for nausea ^35^ and dysgeusia, ^49^ and three articles each for vomiting ^35^^,^^49^^,^^50^ and loss of or decrease in appetite. ^35^^,^^49^^,^^50^

### Prevalence of prolonged GI symptoms in patients with COVID-19

**Figure 2.**
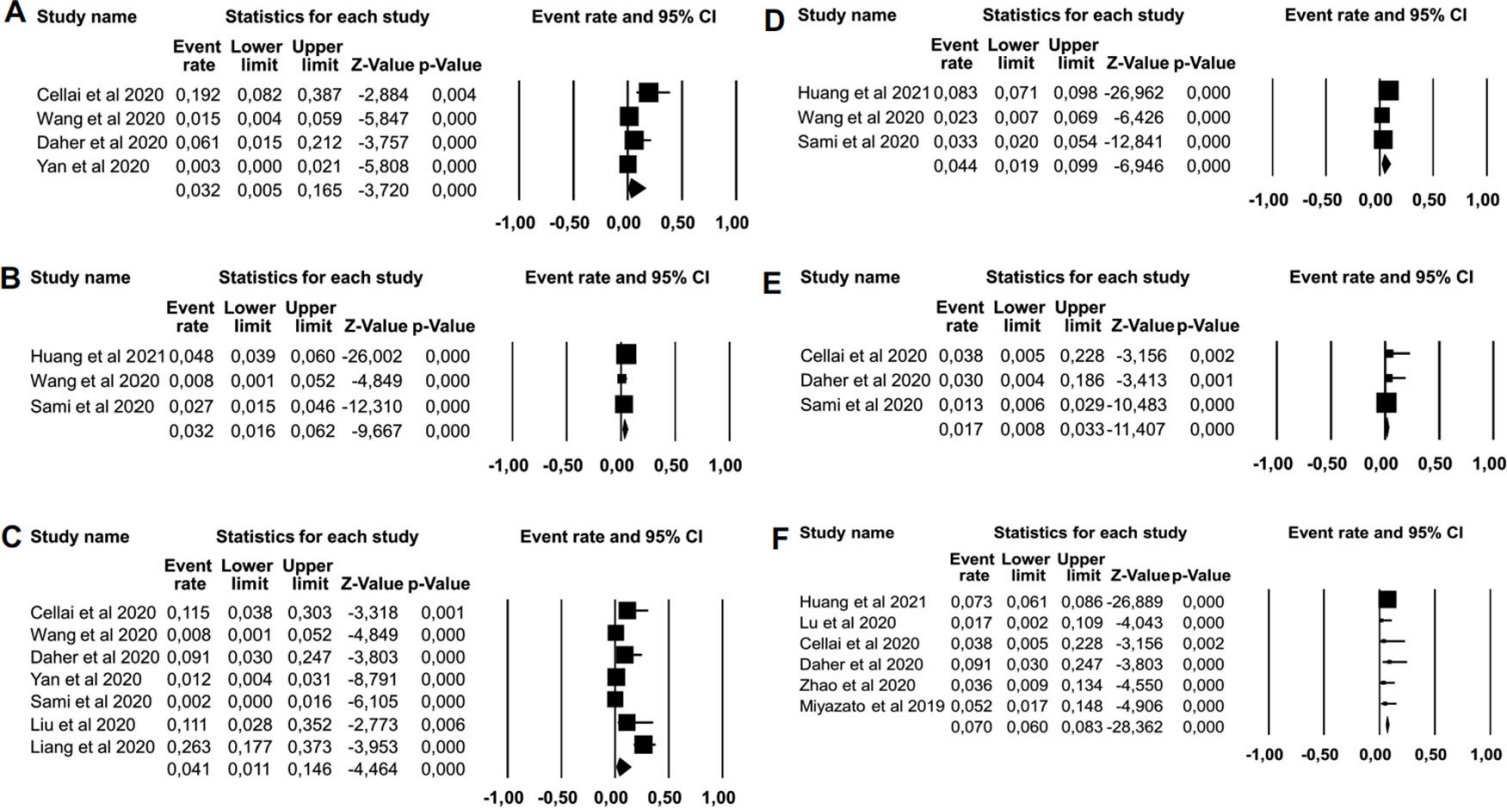
Forest plot of symptoms in long COVID-19 syndrome. (A) Estimated prevalence of prolonged nausea in COVID-19 (event rate 3.23%, 95%CI: 0.54%–16.53%, *p* < 0.001, p Egger 1.683, and p heterogeneity <0.001). (B) Estimated prevalence of prolonged vomiting in COVID-19 patients (event rate 3.19%, 95%CI: 1.62%–6.17%, *p* = 0.028, p Egger 0.482, and p heterogeneity 0.028. (C) Estimated prevalence of prolonged diarrhea in COVID-19 patients (event rate 4.12%, 95%CI: 1.07%–14.64%, *p* < 0.001, p Egger 1.726, and p heterogeneity <0.001). (D) Estimated prevalence of prolonged of loss of appetite in COVID-10 patients (event rate 4.41%, 95%CI: 1.91%–9.94%, *p* < 0.001, p Egger 0.690, and p heterogeneity <0.001. (E) Estimated prevalence of prolonged of abdominal pain in COVID-19 patients (event rate 1.68%, 95%CI: 0.84%–3.32%, *p* = 0.499, p Egger <0.001, and p heterogeneity 0.499). (F) Estimated prevalence of prolonged of dysgeusia in COVID-19 patients (event rate 7.04%, 95%CI: 5.96%–8.30%, *p* = 0.526, p Egger <0.0001, and p heterogeneity 0.052).

Prolonged nausea was reported in 10 patients after recovery, with estimated prevalence of 3.23% (95% CI: 0.54%–16.53%) from a total of 527 patients with COVID-19 from four studies ( Figure 2). Persistent vomiting was identified in 93 of 2,238 patients with COVID-19 from three studies, which corresponded to a pooled prevalence of 3.19% (95% CI: 1.62%–6.17%). Seven articles reported the prevalence of prolonged diarrhea as 4.12% (34/1,073 patients with COVID-19) with 95% CI: 1.07%–14.64% **.** Loss of or decrease in appetite was reported in three studies that included 2,238 patients with COVID-19, among whom 156 patients were reported to have had the symptom (estimated prevalence of 4.41%, 95% CI: 1.91%–9.94%). Based on three studies, abdominal pain was reported in 8/511 patients with COVID-19 with an estimated prevalence of 1.68% (95% CI: 0.84%–3.32%). Six studies identified 130 cases of prolonged dysgeusia among a total of 1,887 patients with COVID-19 (7.04%, 95% CI: 5.96%–8.30%). Abdominal pain and dysgeusia were analyzed using a fixed-effect model of Egger’s test at *p* < 0.001.

### Association of COVID-19 severity and prolonged GI symptoms

Owing to the lack of studies presenting prolonged GI symptoms among patients with mild-moderate and severe COVID-19, the associations were calculated only for vomiting and loss of appetite. The severity of COVID-19 was not associated with the presence of either vomiting or loss of appetite in patients with COVID-19 (odds ratio (OR): 1.19, 95% CI: 0.51–2.78 and OR: 0.84, 95% CI: 0.47–1.5, respectively). Both symptoms were analyzed using the fixed-effect model of Egger’s test at *p* < 0.001.

### Discussion

Studies have confirmed that the existence of SARS-CoV-2 in the GI tract can last for several weeks after a throat swab shows a negative result. ^56^^,^^57^ One study found that the elimination of SARS-CoV-2 from fecal samples was completed more than a month after samples collected from the respiratory tract turned out negative. ^58^ This may explain the prolonged GI symptoms observed in patients with COVID-19 in the present study.

To date, no satisfying explanation is available as to why the virus lasts longer in the gut than in the other systems. Although the exact mechanisms are not fully elucidated, some putative pathophysiological mechanisms underlying the occurrence of COVID-19-induced GI symptoms have been put forward. These potential mechanisms encompass the direct invasion of SARS-CoV-2 in GI cells, secondary effects after other organs are infected, and drug treatment-induced digestive symptoms. ^59^^,^^60^

### Direct invasion of SARS-CoV-2 into GI epithelial cells

**Figure 3.**
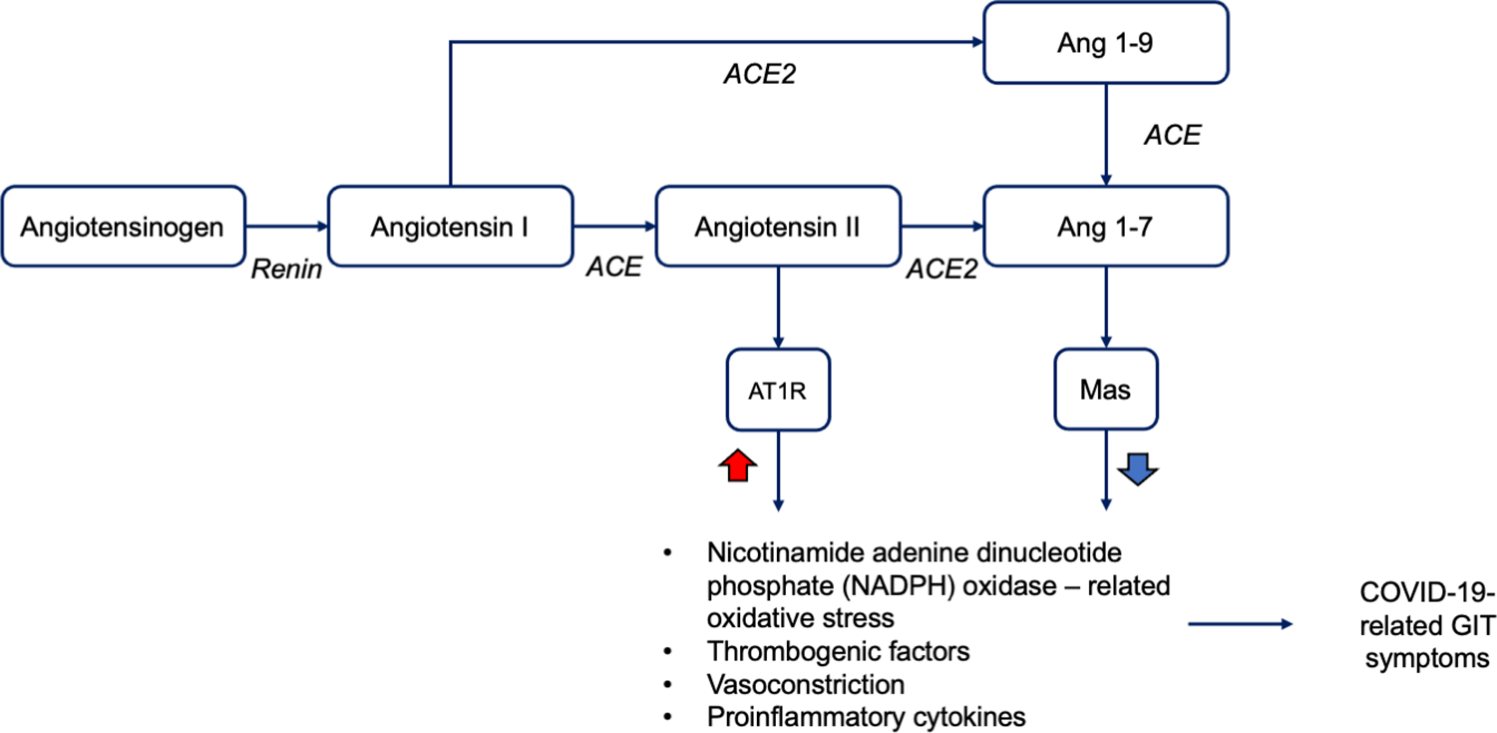
SARS-CoV-2 infection and dysregulation of ACE2/Ang (1–7)/Mas and ACE/Ang II/AT1R axis that are associated with GI tract symptoms. The inactive Ang I is converted into Ang II which produces its biological activities via its binding to AT1R. To maintain the homeostasis status, the catalytic activity of ACE2 converts Ang II to Ang 1-7 which has the opposite action of Ang II through its binding to Mas receptor. The invasion of SARS-CoV-2 to ACE2 causes an accumulation of Ang II and decreased level of Ang 1-7. This condition is linked to the increased interaction between Ang II and its receptor AT1R resulting in the hyperactivity of NADPH oxidase which is related to oxidative stress, massive production of proinflammatory cytokines, increased activity of thrombogenic factors and vasoconstriction. These events are eventually associated with the emergence of the COVID-19 GIT symptoms.

It has been shown that angiotensin-converting enzyme 2 (ACE2), the entry receptor of SARS-CoV-2, is also highly expressed in the digestive system organs such as the esophagus, small intestine, and colon. ^61^^,^^62^ Therefore, it is quite plausible to observe the advent of several GI symptoms induced by SARS-CoV-2 infection in patients ranging from nausea, vomiting, and diarrhea to loss of appetite and abdominal pain. ^59^^,^^60^^,^^63^ The expression of the ACE2 receptor in the gut results in the digestive system also being vulnerable to attack by SARS-CoV-2. After being occupied by the virus, ACE2 becomes dysfunctional, resulting in the impairment of the protective activity of the ACE2/Ang-(1–7)/Mas axis, whereas the activity of the ACE/Ang II/AT1R axis is elevated. ^64^ Following this condition, nicotinamide adenine dinucleotide phosphate oxidases are excessively activated leading to the occurrence of oxidative stress-induced inflammation and this finally causes tissue damage as described in Figure 3. ^65^^,^^66^

Furthermore, when the virus invades the digestive system, the immune cells move to the site of infection and release a massive amount of proinflammatory cytokines, such as interleukin-1β (IL-1β), IL-6, and tumor necrosis factor-alpha (TNF-α) resulting in intestinal inflammation. ^67^ The inflammation of the intestines was confirmed in a study which found that the level of fecal calprotectin, a specific protein biomarker for intestinal inflammation found in the feces, increased in patients with COVID-19. ^68^ Interestingly, the rise in calprotectin is higher in patients who are also suffering from diarrhea ^68^ indicating that diarrhea in SARS-CoV-2 infection might be linked to intestinal inflammation. It has been proposed that intestinal inflammation might cause diarrhea by disturbing the homeostasis of gut microbiota. ^69^ Indeed, several inflammation-related diseases such as ulcerative colitis ^70^ and Crohn’s disease ^71^ are treated using probiotics to overcome gut dysbiosis.

Diarrhea observed in patients with COVID-19 can also be linked to the impairment of the noncatalytic activity of ACE2. The receptor plays a pivotal role in the uptake of neutral amino acids such as tryptophan. ^72^ The uptake of tryptophan into the enterocytes depends on the activity of the B ^0^AT1 transporter which is colocalized with ACE2 to act properly. ^73^ Thus, any dysfunctionalities in ACE2 will perturb the uptake of tryptophan by the cells. Moreover, the disturbances in tryptophan uptake are associated with the decreased activity of the mammalian target of rapamycin (mTOR) signaling pathway which has a responsibility in regulating the expression of antimicrobial peptides secreted by various intestinal cells and this will finally disturb the homeostasis of the gut microbiota. ^72^^–^^75^ By this action, SARS-CoV-2 can induce GI symptoms, such as diarrhea, as observed in patients with COVID-19. ^76^

Furthermore, the suppression of the intestinal commensal microbes can result in worse consequences as these microbes are significantly involved in the regulation of microbiota homeostasis. ^77^ Specifically, in addition to the intestinal cells, antimicrobial peptides, such as short-chain fatty acids (acetate, butyrate, and propionate), can also be produced by the commensals. ^77^ These fatty acids can activate G-protein coupled receptors found in the apical area of the intestinal cells, such as GPR43. ^78^ GPR43 activation is followed by the induction of the mammalian target of rapamycin signaling pathway which has previously been described as the pathway responsible for regulating the expression of antimicrobial peptides, such as defensins and RegIIIγ. ^79^

**Figure 4.**
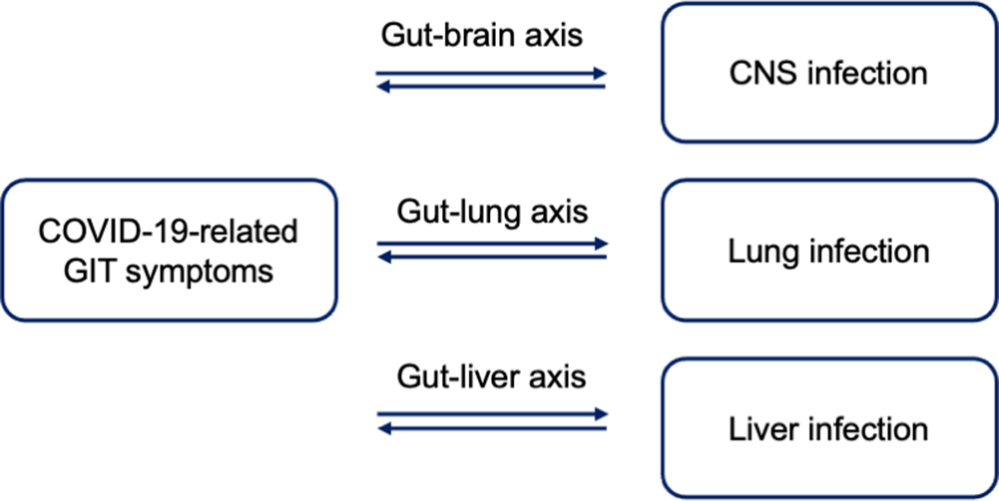
SARS-CoV-2 infection and perturbation of the intestinal flora homeostasis that are associated with GI tract symptoms. The neutral amino acid, such as tryptophan, is taken up by the intestinal cells through the action of an influx transporter B0AT1. To act properly, this transporter works together with ACE2. The activity of ACE2 is independent of RAS system. Once absorbed, tryptophan activates mTOR signaling pathway responsible for the regulation of intestinal antimicrobial peptides expression. During the COVID-19 course, ACE2 is invaded by SARS-CoV-2 disturbing the uptake of the amino acids by B0AT1. This condition is then followed by the inhibition of mTOR pathway resulting in the perturbances of antimicrobial peptides (i.e. defensins and RegIIIγ) secretion into the intestinal lumen. Furthermore, dysbiosis can cause several subsequent effects because it induces the production of proinflammatory cytokines, inhibits anti-inflammatory cytokines, weakens the tightness of the epithelial barrier and decreases the secretion of some beneficial metabolites from the microbiomes. Taken together, these effects result in the emergence of GIT symptoms, such as intestinal inflammation and diarrhea.

Gut microbiomes are also implicated in immune responses where they are found to inhibit the action of proinflammatory cytokines such as IL-1β, IL-6, and TNF-α and to promote the action of anti-inflammatory cytokines such as IL-10. ^77^^,^^78^ Therefore, the imbalance in microbiomes homeostasis could lead to the exacerbation of intestinal inflammation. Another critical role played by the intestinal flora is associated with the regulation of barrier integrity of the intestinal epithelia as it has been found that the flora is involved in the upregulation of tight junction proteins and promotion of mucus secretion. ^77^ Taken together, the perturbation of the intestinal flora homeostasis may induce gut inflammation and promote GI symptoms as observed in patients with COVID-19 ( Figure 4).

The steady-state of gut microbiota could also be impaired by changes in oxygen supply in the intestine as hypoxia is seen in a large portion of patients with COVID-19. ^80^^,^^81^ It has been reported that the microbiome has a critical role in maintaining oxygen levels in the gut to promote the absorption of various nutrients, regulation of the epithelial barrier, and response of the immune system. ^81^

### GI damage as the secondary effect following infection in other organs

**Figure 5.**
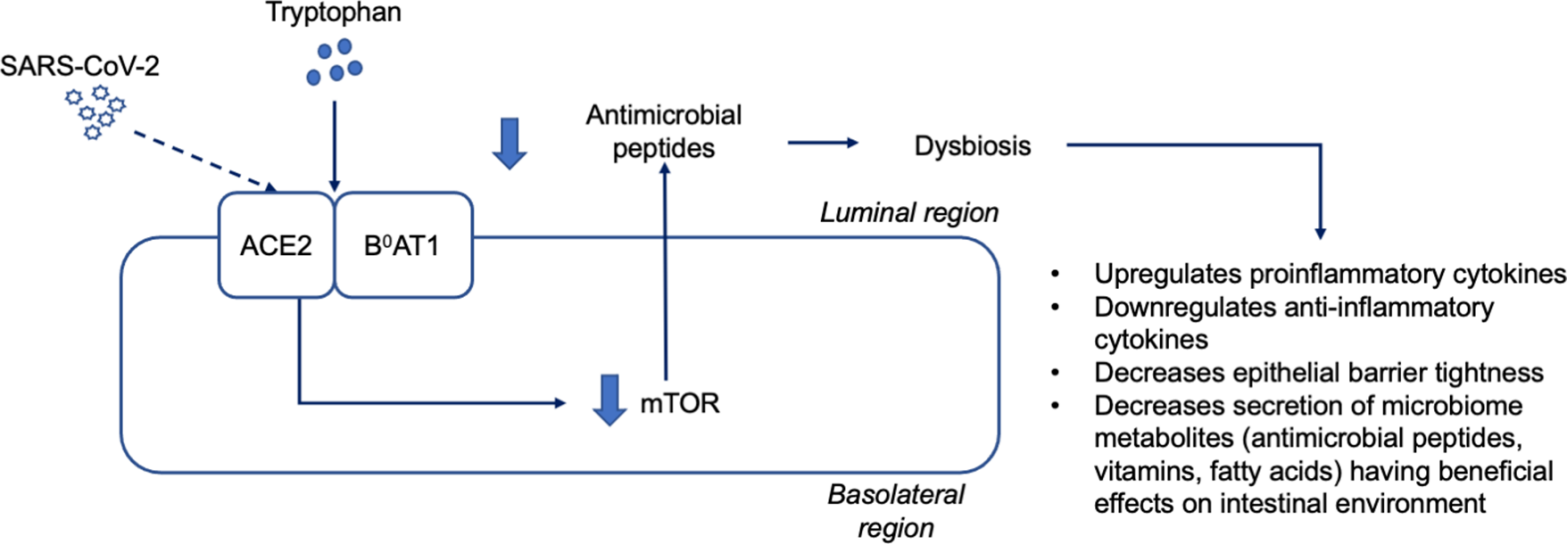
Interrelation between the digestive system and other systems as a proposed mechanism of GI symptoms in SARS-CoV-2 infection. GIT symptoms during the COVID-19 course can be influenced by the virus infections in several sites such as in the CNS, lung and liver. The infections occurring in those organs can be sensed by the GIT leading to the emergence of the symptoms. Several axes are involved in this interrelationship such as the gut-brain axis which is mainly mediated by the enteric nervous system, gut-lung axis involving the movement of CCR9-CD4+ from the lung to the intestine driven by the CCL25, and gut-liver axis which is connected through the portal vein and biliary tract.

The interrelation between the digestive system and other systems or organs inspires another proposed mechanism underlying the emergence of GI symptoms during COVID-19 infection ( Figure 5). As SARS-CoV-2 is not always detected in the feces of patients with COVID-19 who are also displaying GI symptoms, a study speculated that the symptoms are not usually linked to the direct invasion of the virus into the intestines. ^59^ For example, through the gut-lung axis, dysbiosis in the gut is putatively linked to the disturbances in the respiratory flora and vice versa. ^59^^,^^82^^,^^83^ It has been reported that the level of lung-derived C-C chemokine receptor type 9 (CCR9), a chemokine receptor required by CD4 ^+^ to move to the small intestine, increases during respiratory influenza virus infection. ^84^ The movement of CCR9-CD4 ^+^ T cells to the intestine is promoted by the high abundant expression of CCL25 in the small intestine. ^59^^,^^85^ CCL25/CCR9 is found to have a critical responsibility in directing the recruitment of lymphocytes to the small intestine which is subsequently followed by the disruption of intestinal flora homeostasis. ^59^^,^^86^

Several studies suggest that the central nervous system could also be affected by SARS-CoV-2 in addition to the respiratory system as the main system invaded by the virus. ^87^^,^^88^ The infection in the CNS might also affect the digestive system as patients with COVID-19 display some neurological-related GI symptoms, such as nausea and vomiting, without SARS-CoV-2 detection in their stool. ^59^^,^^89^ Conversely, the gut-brain axis might also provide another entry route for the virus to reach the brain. ^89^ The gut-brain axis is connected by the enteric nervous system which is a unique autonomous nervous system because it has both sensory and motor properties. ^90^ Although most of its neurons are not directly innervated by the CNS, critical reciprocal communication occurs between the CNS and the GI system through the enteric nervous system. ^90^ Thus, there is speculation that the involvement of the enteric nervous system as a bridge allows bidirectional passage of either the virus or proinflammatory cytokines. ^83^

Finally, the gut-liver axis must not be overlooked. The invasion and replication of SARS-CoV-2 in the intestine can weaken the epithelial barrier and cause leakage in the gut-blood barrier resulting in the spread of either the virus or its metabolites systemically. ^83^ Junctional proteins and the extracellular matrix as critical components of most barriers in the body, including the gut-blood barrier, ^91^ could be impaired by proinflammatory cytokines. ^66^^,^^92^^–^^95^ The weakening of the barrier could also pave the way for the intestinal flora to reach the liver via the portal vein. ^59^ In turn, through the biliary tract, the liver, supported by cholangiocytes, could transfer microbial metabolites and cytokines into the gut system ^59^^,^^96^ which may eventually initiate the GI symptom. Interestingly, one study speculates that retrograde movement of the virus to the liver through the biliary tract should also be taken into account. ^97^

### Drug-induced gastrointestinal symptoms

The various drugs administered during the COVID-19 course could be linked to the emergence of GI symptoms as adverse reactions. As shown in antimicrobial agents, antiviral agents can also change the steady-state level of the gut microbiota which can cause diarrhea. ^59^ Some antibiotics, such as cephalosporins, penicillins, quinolones, and macrolides are known to induce diarrhea when they are used to treat infections. ^59^^,^^98^ During COVID-19 infection, the use of these antibiotics is common, and has been correlated with the increased number of drug-induced diarrhea cases. ^59^^,^^99^^,^^100^ In general, pharmacological agents can cause diarrhea via a number of mechanisms such as by disturbing the normal flora that reside in the gut, promoting the growth of pathogenic microbes, inducing allergic or toxic reactions in the intestinal mucosa, or by stimulating the motility of the gut. ^101^ In particular, the use of broad-spectrum antibiotics such as penicillins and cephalosporins, is found to be one of the causes of *Clostridium difficile* hegemony over the normal intestinal microbiota. ^102^^,^^103^ This occurs as the antimicrobial agents could kill the flora while leaving the pathogenic microorganisms without control from the normal flora. ^101^

Several antiviral agents are also reported to have GI symptoms as their adverse effects when administered to patients with COVID-19. The use of remdesivir, lopinavir, and ritonavir was found to induce nausea and vomiting. ^104^ The increased level of noxious chemicals, including drugs, in the GI tract could send a signal to the vomiting center in the CNS through afferent fibers of the glossopharyngeal and vagal nerves to induce emesis. ^105^

## Conclusion

Although the pooled prevalence of prolonged GI symptoms in COVID-19 survivors is low, this study adds new insights to the long-term impact of COVID-19 in recovered patients. This systematic review will help increase awareness among clinicians regarding potentially prolonged consequences of COVID-19. Follow-up cohort studies should be designed and managed to identify the effect of this pandemic on the quality of life of the survivors.

## Data availability

### Underlying data

All data underlying the results are available as part of the article and no additional source data are required.

### Reporting guidelines

Figshare: PRISMA checklist for ‘Global prevalence of prolonged gastrointestinal symptoms in COVID-19 survivors and potential pathogenesis: A systematic review and meta-analysis’, https://doi.org/10.6084/m9.figshare.14083613. ^106^

Data are available under the terms of the Creative Commons Attribution 4.0 International license (CC BY 4.0).
