# Supplementary material for: Global prevalence of prolonged gastrointestinal symptoms in COVID-19 survivors and potential pathogenesis: A systematic review and meta-analysis
Source: F1000Res. 2021 Apr 19;10:301. [Version 1] doi: 10.12688/f1000research.52216.1 (PMC8171196; doi:10.12688/f1000research.52216.1)
Supplement: Peer review history

# Peer review: Reviewer response for version 1

**DOI:** 10.5256/f1000research.55461.r85353

**Published:** 2021-05-24

**Authors:** Mahir Gachabayov

**Affiliations:** 1 Department of Surgery, New York Medical College, Valhalla, NY, USA

This systematic review and meta-analysis is aimed at evaluating the prevalence of prolonged GI symptoms and their associations with adverse outcomes among patients with COVID-19. Moreover, the authors aimed to describe possible pathogenetic mechanisms behind such symptoms.

 The concept and the research question make sense. The abstract reflects the core of the findings and is well-written. 

 Introduction is comprehensible and easy to read, describes the gap in the current literature. The objective is stated clearly.

 Methods are adequate. The report complies with the PRISMA guidelines. The protocol of this systematic review was a priori registered in PROSPERO, a fact that mitigates the risks of reporting bias. Eligibility criteria were predefined and make sense. The term 'prolonged symptoms' was defined clearly, a fact that mitigates the risks of detection bias. Endpoints were defined clearly and are clinically relevant. Data sources and search strategy used were comprehensive and the details were reported, which makes the search reproducible. Quality assessment and statistical analysis were comprehensive.

 Results are well-written. PRISMA flow diagram shows nicely the flow of screening and study selection. Table 1 nicely summarizes included studies and Figure 2 nicely depicts the findings of the statistical analysis in forest plots.

 Discussion is comprehensible and easy to read. The findings of this study were discussed in the context of the current evidence. Pathogenesis of the impact of SARS-CoV-2 on the GI tract was well-described alluding to the relevant literature.

 The Conclusion is justified by the findings.

 I have a few comments:

1. I think, the term 'COVID-19 patients' rather than "COVID-19 survivors' should be used. The fact of the matter is that GI symptoms are a part of COVID-19 symptoms. In addition, mortality of GI symptoms was not evaluated to be 0%. Otherwise, the term 'COVID-19 respiratory disease survivors' may be used.
2. Did the included studies discriminate and compare those who primarily had respiratory symptoms and those who had isolated GI disease? Please comment in the Discussion.
3. I compliment the authors for recognizing and commenting on drug-induced GI symptoms. In fact, this entity may have confounded with the statistical findings. And in fact, this could be a reason behind considerable heterogeneity in primary endpoints.
4. Please add a brief paragraph to the end of the Discussion acknowledging the strengths and limitations of the systematic review.
5. Table 1, Study Design: Please change the term 'Cohort' to 'Retrospective' to avoid confusion as prospective observational studies are also cohort studies.

Are the rationale for, and objectives of, the Systematic Review clearly stated?

Yes

Is the statistical analysis and its interpretation appropriate?

Yes

Are sufficient details of the methods and analysis provided to allow replication by others?

Yes

Are the conclusions drawn adequately supported by the results presented in the review?

Yes

Reviewer Expertise:

Clinical outcomes research and evidence synthesis

I confirm that I have read this submission and believe that I have an appropriate level of expertise to confirm that it is of an acceptable scientific standard.

# Peer review: Reviewer response for version 1

**DOI:** 10.5256/f1000research.55461.r83534

**Published:** 2021-05-05

**Authors:** Ari Fahrial Syam

**Affiliations:** 1 Division of Gastroenterology, Department of Intestinal Medicine, Faculty of Medicine, University of Indonesia, Jakarta, Indonesia

I think that this meta-analysis is well done with the PRISMA guidelines. The quality of eligible studies from this meta-analysis was assessed using the Newcastle-Ottawa scale. The protocol of this study was also registered in PROSPERO, an international prospective register for systemic review.

 The topics about GI symptops in COVID-19 survivors are also interesting and need to be of concern to clinicians.

 The 22 studies that were eventually used in this study can be used to draw conclusions about the prevalence of GI symptoms that appear. The pathophysiological explanation for the occurrence of symptoms also makes sense.

 I think this article will benefit for clinician and community.

Are the rationale for, and objectives of, the Systematic Review clearly stated?

Yes

Is the statistical analysis and its interpretation appropriate?

Yes

Are sufficient details of the methods and analysis provided to allow replication by others?

Yes

Are the conclusions drawn adequately supported by the results presented in the review?

Yes

Reviewer Expertise:

Gastroenterology, GERD, H pylori, GERD and COVID-19, Endoscopy and COVID-19

I confirm that I have read this submission and believe that I have an appropriate level of expertise to confirm that it is of an acceptable scientific standard.
